# Large pseudoangiomatous stromal hyperplasia complicated with gynecomastia and lobular differentiation in a male breast

**DOI:** 10.1186/s40064-015-1083-7

**Published:** 2015-06-19

**Authors:** Akiko Mizutou, Kazutaka Nakashima, Takuya Moriya

**Affiliations:** Department of General Surgery, Kawasaki Medical School, 2-1-80 Nakasange, Kita-ku, Okayama, 700-8505 Japan; Pathology 2, Kawasaki Medical School, 577 Matsushima, Kurashiki, Okayama 701-0114 Japan

**Keywords:** Pseudoangiomatous stromal hyperplasia (PASH), Gynecomastia, Lobular differentiation

## Abstract

Pseudoangiomatous stromal hyperplasia (PASH) is a benign lesion often observed in parts of the mammary stroma in a variety of breast diseases. It is characterized by stromal myofibroblastic proliferation that possesses irregularly anastomosing slit-like pseudovascular spaces lined by a layer of spindle cells. PASH commonly occurs in premenopausal women; however, it has also been observed in men with gynecomastia. Although tumor-forming lesions are rare, we report on a case with a large PASH in a male breast complicated with gynecomastia. Imaging showed a tumor with a diameter of >10 cm in the left breast, and aspiration cytology revealed a benign lesion. Since the tumor was large and gradually increased in size, a simple mastectomy was performed. The tumorous lesion was diagnosed as PASH based on the pathological and immunohistological findings. It was complicated by gynecomastia with acinar and lobular formation, which resembled female mammary gland secretory activity that is observed during lactation.

## Background

Pseudoangiomatous stromal hyperplasia (PASH) is a lesion often observed in parts of the mammary stroma in a variety of breast diseases. It is a benign lesion formed by a stromal myofibroblastic proliferation, possessing irregularly anastomosing slit-like pseudovascular spaces lined by a layer of spindle cells (Lakhani et al. 2012). It is sometimes observed as an incidental finding, although tumor-forming lesions are rare. PASH commonly occurs in premenopausal women; however, it has also been reported in postmenopausal women undergoing hormone replacement therapy or men with gynecomastia (Badve and Sloane 1995; Milanezi et al. 1998). It is believed that abnormal reactions to hormones by myofibroblasts are an important factor in its occurrence (Powell et al. 1995).

However, gynecomastia is a common disease in male breasts. Most patients presenting with gynecomastia will have idiopathic, physiological, or secondary gynecomastia due to drugs, liver dysfunction, or an endocrine disorder. The origin of gynecomastia is thought to be closely related to a hormonal imbalance, in which there is a relative excess of estrogen compared to androgen (Braunstein 1993). Pathological images are characterized by prominent epithelial proliferation in ducts and collagenous stroma; acinar and lobular formation is not usually observed.

We report on a large PASH in a male complicated with gynecomastia and lobulation that presented with secretory changes resembling lactation.

## Case report

A 50-year-old man had an ulcer on his lower extremity while receiving outpatient treatment for diabetes and dialysis. He was diagnosed with diabetic gangrene and arteriosclerosis obliterans of the lower limbs. A left breast tumor was discovered upon admission, so he was referred to our department. The patient became aware of the tumor about 1 year ago, and although it gradually increased in size, he never sought medical attention.

A 12 × 11 cm tumor extended over the entire left breast and was palpable. The tumor was elastic, hard, well-defined, and had a smooth surface. No axillary lymph node enlargement was observed.

The blood examination before dialysis showed the following: alanine aminotransferase, 2 U/L; aspartate aminotransferase, 11 U/L; and γ-glutamyl transpeptidase, 12 U/L. The hepatitis B antigen and hepatitis C virus antibody tests were negative. However, anemia was present: red blood cell, 2.48 million/μL; hemoglobin, 7.0 g/dL; hematocrit, 22.7%; and hemoglobin A1c, 6.8%. The kidney function test revealed the following: creatinine, 8.41 mg/dL; and blood urea nitrogen, 29 mg/dL. The patient had no liver dysfunction.

The blood hormone levels before dialysis were as follows: estradiol (E2), 41 pg/mL (our institution’s reference value: 15–35 pg/mL); progesterone, 1.68 ng/mL (0.88–0.00 ng/mL); testosterone, 3.68 ng/mL (8.71–1.31 ng/mL); follicle stimulating hormone (FSH), 35.89 mIU/mL (8.30–2.00 mIU/mL); and prolactin, 76.2 ng/mL (12.8–3.6 ng/mL).

**Figure 1:**
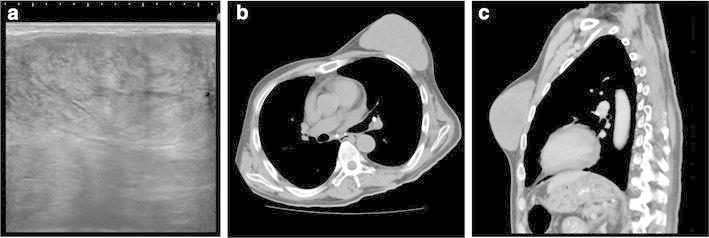
Imaging examination shows a tumor in the left breast. Ultrasonography shows an oval and low-echo tumor, with a heterogeneous internal echo, that possessed slit-like spaces (**a**). Computed tomography shows a well-defined lobular tumor with a maximum diameter of 11 cm in the left breast. **b** Transaxial section; **c** sagittal section.

Ultrasound showed an oval, well-defined and rough, low-echo tumor with somewhat of a heterogeneous internal echo (Figure 1a). Vascularity was low. Computed tomography (CT) showed a well-defined lobular tumor with a maximum diameter of 11 cm in the left breast with no signs of invasion of the chest wall (Figure 1b, c). Two years prior, tumorous lesions could not be confirmed on chest CT. Aspiration biopsy cytology showed a small number of ductal epithelial cells against a background of stromal cells; however, no atypical cells were observed, and a biphasic luminal and myoepithelial pattern was maintained. Additionally, there was an increased cell density of stromal components with some presenting with a spindle shape.

A simple mastectomy was performed under general anesthesia. The tumor was well-defined with no invasion of the surrounding tissue.

**Figure 2:**
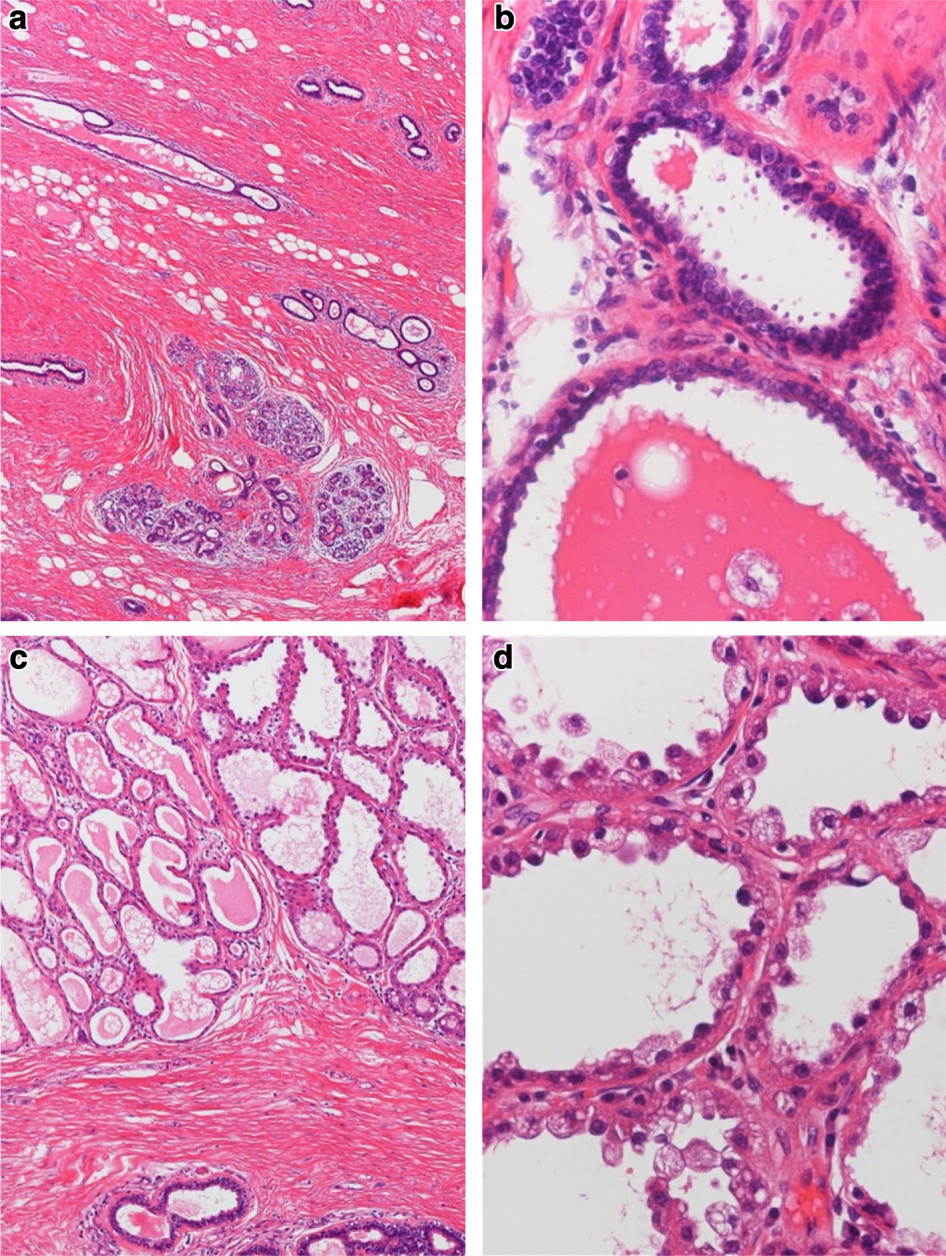
The background demonstrates gynecomastia, characterized by the proliferation of ductal epithelium and stroma (**a** low-power field). There are no atypical cells, and a biphasic pattern is maintained (**b** high-power field). Lobular formation is observed in the area exhibiting gynecomastia (**c** low-power field). The acinar cells contain numerous vacuoles, and appeared to exhibit secretory activity during the lactation period (**d** high-power field).

Pathologically, the background consisted of gynecomastia characterized by epithelial proliferation and stromal fibrosis (Figure 2a). Scattered dilated mammary ducts were observed, sometimes overlaid by ductal epithelium. Signs of hyperplasia such as papillary proliferation were observed; however, there were no atypical cells, and a biphasic pattern was maintained (Figure 2b). Moreover, lobular formation was observed in the gynecomastia. In some, the cytoplasm of the acinar epithelium exhibited vacuolated changes, resembling female mammary glands during the lactation period (Figure 2a, c, d).

**Figure 3:**
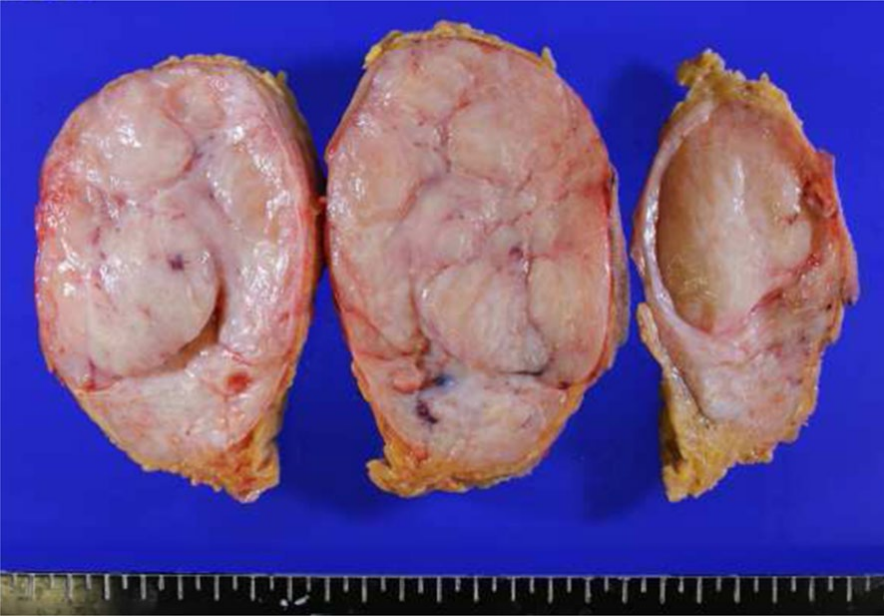
The tumor is well-defined, solid, and lobular with *grayish-white* cross sections.

**Figure 4:**
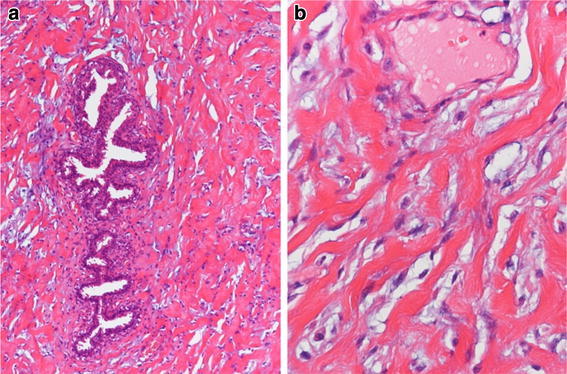
Stromal fibrotic proliferation, with anastomosing slit-like pseudovascular spaces, that is lined with spindle cells. **a** Low-power field; and **b** high-power field.

The tumor of the left breast was well-defined, solid, and lobular with grayish-white cross sections (Figure 3). Pathologically, there was collagenous stromal proliferation intermixed with lobular and ductal elements. The stroma was dense and contained a pseudoangiomatous structure with irregularly anastomosing slit-like spaces. These slit-like spaces were lined by spindle-shaped cells, which presented with mild nuclear hypertrophy, but there was no nuclear division or atypia (Figure 4a, b).

Immunohistologically, epithelial cells of the tumorous area were estrogen receptor (ER) positive and progesterone receptive (PR) positive. Spindle cells showed ER negative, PR negative, vimentin positive, CD34 positive, smooth muscle actin negative, D2-40 negative, CK14 negative, c-kit negative, and type-IV collagen negative. The Ki-67 positive rate of the spindle cells was <5%. Therefore, we diagnosed the tumorous area as PASH.

## Discussion

In 1986, Vuitch et al. reported on a benign disease presenting with a proliferative lesion of the stroma with pseudovascular spaces lined by spindle cells, proposing the concept of pseudoangiomatous hyperplasia of mammary stroma (PHMS) (Vuitch et al. 1986). These lesions reportedly existed in areas of the stroma, which were usually discovered by chance in tissue specimens, and they were found in 23% of biopsy and mastectomy specimens (Ibrahim et al. 1989). Later, the term PASH was proposed for tumoriform lesions (Powell et al. 1995). Lesions that form tumors are also called nodular PASH or tumoriform PASH. PASH commonly occurs in premenopausal women (mean age, 37 years) (Powell et al. 1995). However, it is also often observed in postmenopausal women undergoing hormone replacement therapy, infants (Shehata et al. 2009), and men. Particularly, in men with gynecomastia, it reportedly exists in 98% of PASH cases (Badve and Sloane 1995). Various sizes have been reported from 1 to 12 cm (Jaunoo et al. 2011), with most being about 3–5 cm. The tumor in our patient with macroscopic PASH characteristics had a maximum diameter of 11 cm, but we could find no other reports of such a large tumor in a man.

Histologically, there is a mix of ductal components and fibrotic stroma, but there is usually lack of atypia. When an epithelial lesion is attached, it is often accompanied by varying degrees of hyperplasia (Zanella et al. 1998) or changes that resemble blunt duct adenosis (Moriya et al. 2001). The stroma shows irregularly anastomosing spaces lined with a layer of spindle cells. The stroma around the spaces is formed from a proliferation of hyalinized collagen, which sometimes appears like fibroadenoma entwining epithelial components (Moriya et al. 2001). The spindle cells resemble vascular endothelial cells, but their lumens are void of blood or cellular components, which makes them distinguishable from true blood vessels. Immunohistologically, the spindle cells are positive for vimentin (Ibrahim et al. 1989), positive for smooth muscle actin and CD34 to varying degrees, and are thought to be derived from myofibroblasts (Lakhani et al. 2012; Powell et al. 1995; Zanella et al. 1998).

Since this disease is commonly found in premenopausal women, postmenopausal women undergoing hormone therapy, patients with a history of taking oral contraceptives (Ferreira et al. 2008), and often in men with gynecomastia, its cause is thought to be related to hormone imbalances. Specifically, an abnormal myofibroblast response to hormones is considered important. However, gynecomastia is the enlargement of male mammary glands in response to estrogen (Braunstein 1993). It is caused by a hormone imbalance involving a surplus of estrogen compared to androgen. Gynecomastia occurs in diseases that involve elevated serum estrogen levels, such as estrogen-producing tumors and cirrhosis, which lead to estrogen metabolism disorders and promotion of the production of aromatase due to various reasons. It can also occur in states of relatively high estrogen levels during anti-androgen therapy for prostate cancer (Kumar et al. 2005). Braunstein reported that 25% of gynecomastia cases are idiopathic, 25% are puberty, 10–20% are drug-induced, 8% are due to cirrhosis or malnutrition, 8% accompany primary hypogonadism, 3% occur with testicular tumors, 2% have secondary hypogonadism, 1.5% have hyperthyroidism, and 1% have renal disease (Braunstein 1993). Nagel et al. (1973) reported gynecomastia in 58% of chronic renal failure and dialysis patients, while Vircburger et al. (1985) reported 42%, which are high rates.

The endocrine environment of the hypothalamus-pituitary–gonadal axis is greatly affected by chronic renal failure and dialysis, leading to hypergonadotropic hypogonadism (uremic hypogonadism) (Iglesias et al. 2012). The functions of Leydig cells in the testes of dialysis patients are damaged, causing most patients to have low testosterone levels, normal to mildly high estrogen levels, and normal to high LH/FSH levels. In contrast, an elevated blood prolactin concentration is also reported. This kind of endocrine environment is thought to cause gynecomastia in dialysis patients. Further, dialysis patients are often taking hypotensive agents, anti-gastric ulcer agents, and other drugs that can cause drug-induced gynecomastia, which contributes to the situation. Our case exhibited slightly high estrogen levels and normal testosterone, suggesting a state of elevated estrogen. The patient also had a history of internal Ca-blocker and proton pump inhibitor administration, both of which affect the prolactin concentration and estrogen/androgen ratio. We surmise that these background factors influenced the onset of gynecomastia and the formation of PASH. Moreover, a marked rise in the blood prolactin concentration can stimulate the mammary glands causing pathological formations, which may have produced the lactation period-like lobulation—an extremely rare finding.

Mammary glands are derived from the ectoderm. Their lumens create mammary ducts during the formation and branching of mammary anlagen from the mammary ridge. Initially, the breasts are undeveloped in men and women, and there are no sex differences until puberty. During and after puberty, estrogen causes the mammary ducts in women to grow and lengthen, and estrogen and progesterone promote differentiation of the lobes and acinus. This occurs in the presence of substances such as growth hormones, prolactin, and insulin. However, male mammary glands never develop. Only a few mammary ducts are found below the nipple, and there is generally no lobular structure. Gynecomastia also reflects the normal, lobeless structure; thus, in histopathological findings, a proliferation of ductal epithelium and stromal tissue is characteristic, but a lobular structure is usually not found. However, lobular formation can be observed in male mammary glands due to extraneous hormone administration or other causes. An extensive search for autopsy cases showed that of 65 gynecomastia cases, 28 had prostate cancer with a history of estrogen administration as a part of hormone therapy. Of these, 8 cases (29%) exhibited definite acinus formation, and of those, 2 showed secretory changes that resembled the lactation period in women (Schwartz and Wilens 1963). These two cases had no history of taking progesterone. However, lactation period-like changes to the mammary glands have been reported in a case of sex reversal from male to female (Kanhai et al. 2000). This patient underwent hormone therapy, which consisted of progesterone for chemical castration combined with estrogen for feminization.

Although estrogen is deeply involved in the development of gynecomastia and in the creation of lactation period-like changes in the mammary glands, the involvement of other hormones, including progesterone, androgen, and prolactin cannot be ruled out. In our case, we believe that a complex interaction of numerous hormones developed the pathology for PASH complicated with gynecomastia and lactation period-like changes.

Regarding prognosis, there have been no reports on PASH becoming malignant. The reported recurrence rate ranges from 13 to 26% (Gresik et al. 2010), and local recurrence is frequent if resection is insufficient. Therefore, standard treatment is complete resection by wide excision. In our case, we performed mastectomy of the affected side, and 14 months postoperatively, there were no signs of recurrence.

PASH is a lesion mainly in the mammary stroma that needs to be differentiated from fibroadenoma, phyllodes tumor, and vascular tumors by the presence of vessel-like spaces. Clinically, these lesions tend to grow and are sometimes diagnosed as cancer; therefore, this disease should be considered when diagnosing mammary tumors in men. Although there are no reports of malignancy, patients should be observed closely because of the possibility of recurrence.

## Conclusion

We report on a large PASH in a male breast complicated with gynecomastia. It presented acinar and lobular formation in gynecomastia, which simulated female mammary gland secretory activity that is observed during lactation.

The tumor of PASH had a maximum diameter of 11 cm, but we could find no other reports of such a large tumor in a man. PASH is commonly found in premenopausal women, but it can be observed in men with gynecomastia. It is believed that hormonal imbalance is an important factor in its occurrence.

It is an extremely rare finding that PASH complicated with gynecomastia and lactation period-like change. A history of estrogen administration as a part of hormone therapy is observed in most cases with lactation change. We believe that a complex interaction of numerous hormones developed the pathology for PASH complicated with gynecomastia and lactation period-like changes.

Clinically, these lesions tend to grow and are sometimes diagnosed as cancer; therefore, this disease should be considered when diagnosing mammary tumors in men.
